# Supplementary material for: Mapping the evolution of fertility support policies in China: A content and instrumental analysis
Source: PLoS One. 2025 Oct 9;20(10):e0332137. doi: 10.1371/journal.pone.0332137 (PMC12510515; doi:10.1371/journal.pone.0332137)
Supplement: S1 Appendix — (ZIP) [file pone.0332137.s001.zip › S1 Appendix. 226 original policy documents/186-国家发展改革委办公厅关于做好《国务院办公厅关于促进养老托育服务健康发展的意见》贯彻落实工作的通知(FBM-CLI-4-353509).doc]

国家发展改革委办公厅关于做好《国务院办公厅关于促进养老托育服务健康发展的意见》贯彻落实工作的通知

**发布部门：** [国家发展和改革委员会(含原国家发展计划委员会、原国家计划委员会)](https://www.pkulaw.com/law/chl?Aggs.IssueDepartment=60301) [机构沿革](javascript:void(0))

**发文字号：**发改办社会〔2021〕192号

**发布日期：**2021.02.24

**实施日期：**2021.02.24

**时效性：** [现行有效](https://www.pkulaw.com/law/chl?Aggs.TimelinessDic=01)

**效力级别：** [部门工作文件](https://www.pkulaw.com/law/chl?Aggs.EffectivenessDic=XE0303)

**法规类别：** [老少妇幼残保护](https://www.pkulaw.com/law/chl?Aggs.Category=01604)

国家发展改革委办公厅关于做好《国务院办公厅关于促进养老托育服务健康发展的意见》贯彻落实工作的通知
（发改办社会〔2021〕192号）

中直管理局，教育部、科技部、工业和信息化部、民政部、财政部、人力资源社会保障部、自然资源部、住房城乡建设部、交通运输部、商务部、文化和旅游部、国家卫生健康委、应急部、人民银行、国务院国资委、税务总局、市场监管总局、体育总局、国家统计局、国家医保局、国管局、银保监会、证监会办公厅（室），全国总工会、共青团中央、全国妇联、中国残联办公厅，各省、自治区、直辖市及计划单列市、新疆生产建设兵团发展改革委：
　　为深入贯彻落实《[国务院办公厅关于促进养老托育服务健康发展的意见](https://www.pkulaw.com/chl/d1ad8fb179db9915bdfb.html)》（国办发〔2020〕52号，以下简称《意见》），现就有关事项通知如下：
　　一、深入学习领会《意见》的重要意义
　　养老托育等“一老一小”问题，关系亿万老年人幸福晚年，关系亿万儿童健康成长，关系家庭和社会和谐稳定，关系党和国家事业发展未来。
　　《意见》以习近平新时代中国特色社会主义思想为指导，全面贯彻党的十九大和十九届二中、三中、四中、五中全会精神，坚持问题导向和目标导向，针对当前养老托育服务发展面临的形势和瓶颈制约，系统提出健全政策体系、扩大服务供给、打造发展环境、完善监管服务等4个方面23项改革创新措施，是坚持以人民为中心发展思想的集中体现，是实施积极应对人口老龄化国家战略的关键举措，是服务业提质扩容增效的重要方面，对于减轻家庭养老育幼负担、促进人口长期均衡发展、保障和改善民生、培育经济发展新动能等，都具有重要意义。
　　各地区、各部门要进一步提高认识，抓好《意见》的组织实施，切实落实好促进养老托育服务健康发展各项工作。

　　二、结合实际推进《意见》的贯彻落实
　　为抓好《意见》贯彻实施，国务院办公厅在印发文件的同时，以附件形式明确提出了33项重点任务分工，责任单位涉及28个部委和单位以及地方各级人民政府。
　　各部门要根据重点任务分工，按年度制定工作计划，明确阶段性目标成效、时间节点、具体措施、责任单位、责任人等，确保改革政策措施逐项落地落细。
　　各地区要坚持党委领导、政府主导，将促进养老托育服务健康发展作为一项重要职责和民生工程，纳入经济社会发展规划，列入政府重要议事日程。要对照《意见》提出的政策措施和重点任务，以强化各级党委政府责任、解决发展瓶颈制约等方面内容为重点，结合实际尽快研究制定有针对性、可操作的贯彻落实《意见》工作方案。

　　三、加快建立“一老一小”相关工作机制
　　按照《意见》要求，地方各级政府要加快建立健全“一老一小”工作推进机制，要将建立健全工作机制与研究制定本地贯彻落实《意见》工作方案相结合、与制定“十四五”养老托育专项规划或“一老一小”整体解决方案相结合，着重在组织机构、人员配备、经费投入等方面予以保障，加强与人大等机构的协调配合，建立定期向同级人民代表大会常务委员会报告服务能力提升成效机制。
　　国家发展改革委负责牵头会同各相关部门建立“一老一小”服务能力评价机制，探索开展各地区养老托育服务能力评估，组织交流工作进展情况，加强对《意见》执行情况的跟踪督促，及时向国务院报告《意见》贯彻落实情况，不断促进养老托育服务健康发展。

　　四、营造促进养老托育健康发展的良好社会氛围
　　各地区要积极做好《意见》的宣传解读，主动发布权威信息，及时回应社会关切，凝聚改革共识。要充分利用报刊、杂志、电视、广播等传统媒体和微博、微信、短视频等新媒体开展集中报道，形成宣传工作合力，面向社会公众普及《意见》提出的政策措施，让相关行业和广大群众充分了解“政策红利”。要加强对贯彻落实《意见》过程中的典型经验做法的收集整理，并通过多种方式及时宣传推广，强化示范引领，努力营造全社会重视、支持养老托育服务健康发展的良好社会氛围。
　　各地区、各有关部门请分别于2021年3月底前将本地区贯彻落实《意见》工作方案和本部门2021年度工作计划报送国家发展改革委，并于2021年底前报送2021年度工作落实情况。
　　联系人：崔文通，010-68502590、甘余超，010-68501411
　　传　真：010-68502779

国家发展改革委办公厅
　　2021年2月24日

© 北大法宝：（[www.pkulaw.com](https://www.pkulaw.com/)) 专业提供法律信息、法学知识和法律软件领域各类解决方案。北大法宝为您提供丰富的参考资料，正式引用法规条文时请与标准文本核对。
欢迎查看所有[产品和服务](https://www.pkulaw.com/helps/2.html)。法宝快讯：[如何快速找到您需要的检索结果？](https://www.pkulaw.com/helps/70.html)[法宝](https://www.pkulaw.com/helps/69.html)V6有何新特色？

扫描二维码阅读原文

**原文链接：**<https://www.pkulaw.com/chl/8e21ab9dd20628f0bdfb.html>
